# Intracranial Bleeding After Reperfusion Therapy in Acute Ischaemic Stroke Patients Randomized to Glyceryl Trinitrate vs. Control: An Individual Patient Data Meta-Analysis

**DOI:** 10.3389/fneur.2020.584038

**Published:** 2020-10-20

**Authors:** Jason P. Appleton, Lisa J. Woodhouse, Nikola Sprigg, Joanna M. Wardlaw, Philip M. Bath

**Affiliations:** ^1^Stroke Trials Unit, Division of Clinical Neuroscience, University of Nottingham, Nottingham, United Kingdom; ^2^Stroke, University Hospitals Birmingham National Health Service Foundation Trust, Birmingham, United Kingdom; ^3^Stroke, Nottingham University Hospitals National Health Service Trust, Nottingham, United Kingdom; ^4^Centre for Clinical Brain Sciences, University of Edinburgh, Edinburgh, United Kingdom

**Keywords:** bleeding, glyceryl trinitrate, ischaemic stroke, reperfusion, thrombolysis, thrombectomy, meta-analysis

## Abstract

**Background:** Thrombolysis, with or without thrombectomy, for acute ischaemic stroke is associated with an increased risk of intracranial bleeding. We assessed whether treatment with glyceryl trinitrate (GTN), a nitric oxide donor, may influence the associated bleeding risk.

**Methods:** We searched for completed randomized controlled trials of GTN vs. no GTN in acute ischaemic stroke with data on reperfusion treatments (thrombolysis and/or thrombectomy). The primary efficacy outcome was functional status as assessed by the modified Rankin Scale (mRS) at day 90; the primary safety outcome was intracranial bleeding. Secondary safety outcomes included symptomatic intracranial hemorrhage and haemorrhagic transformation of infarction. Individual patient data were pooled and meta-analysis performed using ordinal or binary logistic regression with adjustment for trial and prognostic variables both overall and in those randomized within 6 h of symptom onset.

**Results:** Three trials met the eligibility criteria. Of 715 patients with ischaemic stroke who underwent thrombolysis (709, >99%) or thrombectomy (24, 3.4%), 357 (49.9%) received GTN and 358 (50.1%) received no GTN. Overall, there was no difference in the distribution of the mRS at day 90 between GTN vs. no GTN (OR 0.94, 95% CI 0.72–1.23; *p* = 0.65); similarly, there was no difference in intracranial hemorrhage rates between treatment groups (OR 0.90, 95% CI 0.43–1.89; *p* = 0.77). In those randomized to GTN vs. no GTN within 6 h of symptom onset, there were numerically fewer bleeding events, but these analyses did not reach statistical significance.

**Conclusions:** In ischaemic stroke patients treated predominantly with thrombolysis, transdermal GTN was safe, but did not influence functional outcome at 90 days.

## Introduction

Thrombolysis with alteplase for acute ischaemic stroke is an efficacious treatment if given within 4.5 h of onset, but is associated with an increased risk of symptomatic intracranial hemorrhage (sICH: 6.8% thrombolysis vs. 1.3% placebo) (1). In the context of large vessel occlusion of the anterior cerebral circulation, thrombectomy is highly effective at improving clinical outcomes when performed within 6 h of onset (2), and upto 24 h in those with perfusion mismatch (3). In trials including patients who received alteplase, there was no difference in sICH rates between those randomized to thrombectomy and no thrombectomy (2). Further, a recent trial assessing tPA + thrombectomy vs. thrombectomy alone, found no difference in sICH rates (4).

Raised blood pressure (BP) in those undergoing thrombolysis has been associated with increased risk of haemorrhagic transformation of infarction (HTI) and sICH. The ENCHANTED-BP trial found that intensive lowering of BP in those undergoing thrombolysis did not improve functional outcome but did reduce the rate of sICH as compared with guideline BP management (5). It is unclear whether specific BP agents exert effects that may augment reperfusion strategies. The nitric oxide donor, glyceryl trinitrate (GTN), has been associated with improved clinical outcomes when administered as a patch within 6 h of stroke onset in a subgroup of the large ENOS trial (6). However, when administered in the pre-hospital setting within 4 h of onset, GTN had a neutral effect on functional outcome (7). Data from a small pilot study in the ambulance found that treatment with GTN was associated with a tendency toward increased rates of thrombolysis compared with no GTN, suggesting that GTN may prime patients for thrombolysis by lowering their BP into the treatment range (8). Little is known about whether GTN influences the bleeding risk associated with reperfusion treatments. Here, we assessed the effect of GTN in those undergoing reperfusion therapies and the associated bleeding risk.

## Methods

The study was registered with PROSPERO (CRD42020193427) and followed PRISMA guidance (Supplementary Table 1).

### Search Strategy and Selection Criteria

We searched EMBASE, PubMed, and Cochrane Library for randomized controlled trials of GTN in adults with acute stroke and data on reperfusion treatments, including thrombolysis or thrombectomy, using the search terms: “stroke” OR “cerebral ischaemia,” AND “glyceryl trinitrate” OR “nitroglycerin,” AND “randomized,” OR “randomized.” Non-randomized studies were excluded. We aimed to perform an individual patient data meta-analysis of the trials found, focusing on acute ischaemic stroke patients treated with reperfusion strategies. Participants with available data on reperfusion therapy and modified Rankin Scale (mRS) score at 90 days were eligible for inclusion.

Search results were screened, abstracts reviewed and full-text manuscripts assessed for inclusion criteria by one author (JPA). We assessed risk of bias of the included studies using Cochrane's “risk of bias” tool as high, low or unclear risk across the following elements: random sequence generation; allocation concealment; blinding of participants and personnel; blinding of outcome assessment; incomplete outcome data; selective reporting; and other sources of bias (9).

The chief investigator of the included studies was approached to share individual patient data for use in the meta-analysis. Ethical approval was obtained for each of the included trials and informed consent was received from participants or their legal representative.

### Clinical Outcomes

The primary efficacy outcome was functional outcome measured using the mRS at day 90; a seven level ordinal scale ranging from 0 = no symptoms, through increasing levels of dependency, to 6 = death.

The primary safety outcome was intracranial hemorrhage; secondary safety outcomes focused on bleeding using accepted definitions of sICH, HTI, extracranial bleeding (ECB), and major extracranial hemorrhage (MEH). In the included trials, imaging data were collected at sites and adjudicated centrally by trained neuroradiologists using a set proforma (10). Safety data on death were also collected at day 7 and day 90.

Outcomes were assessed overall across the total population of the included trials, and in those participants randomized to GTN vs. no GTN within 6 h of stroke onset in order to assess the effect of GTN in close proximity to reperfusion therapies.

### Data Analysis

The individual patient databases of the included trials were merged by LJW. Participants with a non-ischaemic stroke diagnosis, or those who did not receive reperfusion therapies, were removed from the dataset. Data on recruitment, age, sex, baseline mRS, prior medical problems, baseline National Institutes for Health Stroke Scale (NIHSS), stroke etiology, stroke syndrome, stroke type, time to randomization, baseline hemodynamics, mRS at day 90, death at day 7, and 90, intracranial bleeding, HTI, sICH, ECB, and MEH were extracted.

Data are number (%), mean (standard deviation, SD), median [interquartile range]. Baseline characteristics were compared across trials by Chi-square test, Kruskal–Wallis test, or one-way ANOVA as appropriate. Data between treatment groups were assessed by intention-to-treat. Ordinal or binary logistic regression was used to assess differences in clinical outcomes between treatment groups with resultant odds ratio (OR) with 95% confidence intervals (CI) provided. Analyses were adjusted for prognostic variables including age, sex, baseline mRS, baseline NIHSS, baseline systolic BP, time to randomization, and trial. Subgroup analyses were performed by adding an interaction term to an unadjusted ordinal logistic regression model. Significance was set at *p* < 0.05. Analyses were performed using Statistical Package for the Social Sciences (SPSS) version 23.

## Results

### Included Studies

Three trials met the inclusion criteria: RIGHT (8), ENOS (11), and RIGHT-2 (7) trials (Supplementary Figure 1). All three trials assessed transdermal GTN 5 mg patches vs. no patch, but varied in design and delivery. RIGHT (*n* = 41) and RIGHT-2 (*n* = 1149) were both pre-hospital paramedic-delivered trials that randomized patients with presumed stroke to transdermal GTN or no GTN within 4 h of onset. There were 6 further days of trial treatment in hospital in RIGHT and 3 further days in RIGHT-2. In contrast, ENOS (*n* = 4,011) was a large in-hospital trial that randomized participants with stroke within 48 h of onset to GTN or no GTN given for 7 days. Therefore, patients in RIGHT and RIGHT-2 received GTN or no GTN in the ambulance prior to reperfusion therapies in hospital, whilst most ENOS participants with ischaemic stroke were randomized after the thrombolysis window. All participants underwent baseline neuroimaging. Follow-up imaging was stipulated as part of the RIGHT-2 trial at day 2 and as clinically indicated in all three trials e.g., 24 h post-thrombolysis/thrombectomy. All neuroimaging scans were adjudicated centrally by trained neuroradiologists. All participants had data extracted for the clinical outcomes specified, where available. Further details pertaining to the conduct and results of the individual trials are published (7, 8, 11).

### Risk of Bias of Included Studies

Full details are provided in a “risk of bias” table (Supplementary Table 2) and associated figures (Supplementary Figures 2, 3). In summary, all three trials had low risk of bias in relation to the elements assessed except for blinding of participants and personnel (performance bias), which was an unclear risk of bias in all three included studies. There was no placebo patch available in any of the included studies and therefore a gauze dressing was applied over the GTN patch or equivalent area of skin in ENOS (11) and RIGHT (8), whilst in RIGHT-2 (7) a Duoderm dressing was placed on the skin of those randomized to no GTN with a gauze dressing on top. As such, participants were blinded but investigators applying the patch/dressing were not.

### Baseline Characteristics

Data were available for 715 participants (GTN 357, no GTN 358) with acute ischaemic stroke who underwent reperfusion therapies from the three included studies. Data on reperfusion treatment were available in 414 patients recruited into ENOS (from 2003 to 2013), 10 patients in RIGHT (2010–2011) and 291 patients in RIGHT-2 (2015–2018) (Table 1). Overall, the average age was 72 years old, 45% were female, 62% had hypertension, and 21% atrial fibrillation (AF). The mean NIHSS score was 12.5 and median GCS 15. A substantial proportion of participants (36%) were deemed to have a cardioembolic etiology for their stroke. Baseline BP was 162 / 87 mmHg, with a median time to randomization of 4.9 h. The vast majority underwent thrombolysis (709, 99%) with 24 (3.4%) patients being treated with thrombectomy. Of the 24 participants who underwent thrombectomy, six did not receive thrombolysis (Table 1).

**Table 1:** Baseline characteristics of ischaemic stroke participants who underwent reperfusion strategies by trial.

	**All**	**ENOS**	**RIGHT**	**RIGHT-2**	***p***
Number of patients	715	414	10	291	
Years of recruitment, range	2003–2018	2003–2013	2010–2011	2015–2018	–
Median, [IQR]	2013 [2011, 2017]	2012 [2010, 2012]	2010 [2010–2011]	2017 [2016–2017]	<0.001
Age (years)	72.4 (11.6)	71.0 (11.4)	78.2 (5.9)	74.2 (11.8)	<0.001
Sex, male (%)	393 (55.0)	239 (57.7)	7 (70.0)	147 (50.5)	0.10
mRS [/6]	0 [0, 1]	0 [0, 0]	0 [0, 1]	0 [0, 1]	<0.001
Medical history (%)					
Hypertension	444 (62.1)	265 (64.0)	7 (70.0)	172 (59.1)	0.37
Diabetes mellitus	118 (16.5)	57 (13.8)	2 (20.0)	59 (20.3)	0.07
Atrial fibrillation	136 (20.6)	73 (17.6)	2 (20.0)	61 (25.7)	0.048
Stroke	88 (12.3)	43 (10.4)	1 (10.0)	44 (15.1)	0.17
TIA	92 (13.1)	53 (13.2)	3 (30.0)	36 (12.4)	0.27
IHD	103 (14.5)	59 (14.5)	0	44 (15.1)	0.41
PAD	22 (3.1)	18 (4.5)	0	4 (1.4)	0.058
Smoking, current	139 (19.4)	85 (20.5)	4 (40.0)	50 (17.2)	0.14
Alcohol >21 units per week	51 (7.1)	33 (8.0)	0	18 (6.2)	0.45
Qualifying event (%)					
Ischaemic stroke	714 (99.9)	414 (100.0)	10 (100.0)	290 (99.7)	0.48
TIA	0	0	0	1 (0.3)	0.48
NIHSS (/42)	12.5 (6.0)	12.2 (5.3)	11.7 (5.3)	13.0 (7.0)	0.17
GCS [/15]	15 [13,15]	15 [14,15]	15 [13,15]	14 [12,15]	<0.001
TOAST classification (%)					
Cardioembolic	252 (35.7)	136 (32.9)	5 (50.0)	111 (39.4)	0.14
Large vessel	157 (22.2)	106 (25.6)	2 (20.0)	49 (17.4)	0.037
Small vessel	121 (17.1)	85 (20.5)	1 (10.0)	35 (12.4)	0.017
Other	179 (25.4)	88 (21.3)	2 (20.0)	89 (31.6)	0.008
Hemodynamics					
BP, Systolic (mmHg)	161.6 (19.2)	163.1 (16.0)	176.5 (27.7)	158.9 (22.4)	0.001
BP, Diastolic (mmHg)	87.2 (14.1)	85.9 (12.4)	92.5 (22.8)	88.7 (15.9)	0.019
Heart rate	78.8 (18.5)	76.6 (15.7)	84.7 (22.6)	81.8 (21.3)	0.001
Time to randomization [hours]	4.9 [1.1, 23.5]	21.7 [7.6, 28.7]	1.1 [0.7, 2.1]	1.0 [0.7, 1.4]	<0.001
Thrombolysis (%)	709 (99.2)	414 (100.0)	10 (100.0)	285 (97.9)	0.012
Thrombectomy (%)	24 (3.4)	-	-	24 (8.5)	-

*Data are number (%), mean (standard deviation, SD), median [interquartile range, IQR]; comparison across trials by Chi-square test, Kruskal–Wallis test, or one-way ANOVA*.

*BP, blood pressure; ENOS, Efficacy of nitric oxide in stroke trial; GCS, Glasgow coma score; IHD, ischaemic heart disease; mRS, modified Rankin Scale; NIHSS, National Institutes for Health Stroke Scale; PAD, peripheral arterial disease; RIGHT, Rapid intervention with glyceryl trinitrate in hypertensive stroke trial; TIA, transient ischaemic attack*.

Across the three trials the participants were older and baseline mRS was higher in RIGHT and RIGHT-2 than in ENOS (Table 1). Similarly, AF rates differed across the trials with a higher proportion in RIGHT-2. Median GCS was lower in RIGHT-2 than ENOS and RIGHT. Large vessel and small vessel stroke etiologies were more common in ENOS than RIGHT or RIGHT-2. Baseline BP and heart rate also varied across the trials. Due to the different randomization periods across the trials, participants were randomized at around 1 h after symptom onset in RIGHT and RIGHT-2, and at 22 h in ENOS. The 24 patients who received thrombectomy were all from the RIGHT-2 trial (Table 1).

### Clinical Outcomes

**Figure 1:**
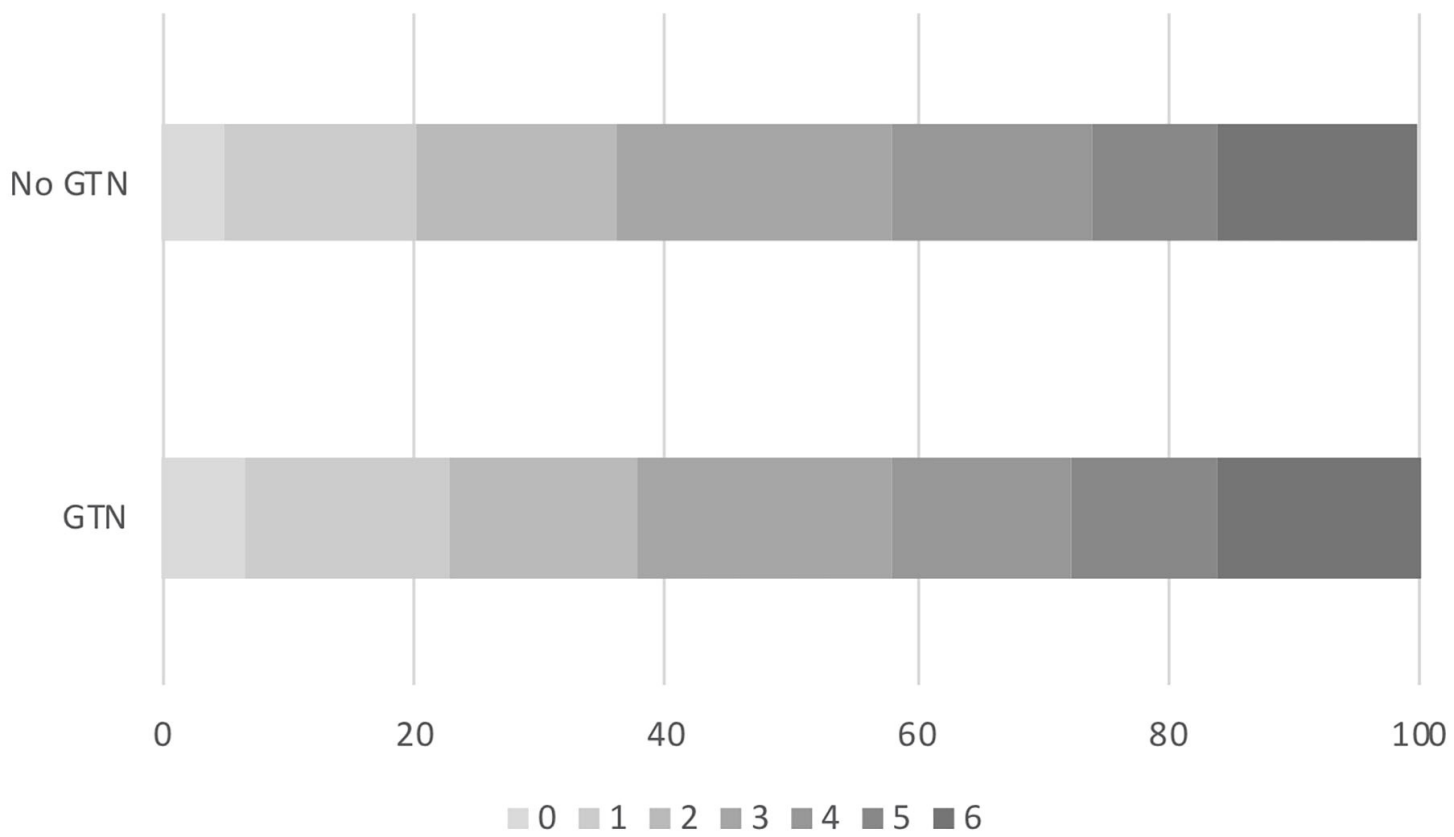
Acute ischaemic stroke patients who underwent reperfusion strategies. Distribution of mRS at day 90 by GTN vs. no GTN: adjusted OR 0.94, 95% CI 0.72–1.23, *p* = 0.65. CI, confidence interval; GTN, glyceryl trinitrate; mRS, modified Rankin Scale; OR, odds ratio.

Overall, the distribution of the mRS at day 90 did not differ by randomization to GTN or no GTN in unadjusted and adjusted analyses: adjusted OR 0.94, 95% CI 0.72–1.23, *p* = 0.65 (Table 2 and Figure 1). There were 32 (4.5%) intracranial bleeds, of which 12 (1.7%) were sICH. There were no differences in bleeding rates between the GTN and no GTN groups. There were numerically fewer HTIs in the GTN than no GTN groups (8 vs. 11) although this was not statistically significant. ECB and MEH were uncommon and did not differ by randomized treatment. No differences between GTN groups were noted for death at day 7 or day 90 (Table 2).

**Table 2:** Clinical outcomes of acute ischaemic stroke patients who underwent reperfusion strategies by GTN vs. no GTN.

**Overall**	**GTN**	**No GTN**	**OR (95% CI)**	***p***
N (%)	357 (49.9)	358 (50.1)		
**Primary efficacy outcome**				
mRS at day 90 [/6]–adjusted	3 [2,5]	3 [2,5]	0.94 (0.72, 1.23)	0.65
Unadjusted			0.96 (0.74, 1.24)	0.74
**Primary safety outcome**				
Intracranial hemorrhage (%)	15 (4.2)	17 (4.7)	0.90 (0.43, 1.89)	0.77
**Secondary safety outcomes**				
sICH (%)	6 (1.7)	6 (1.7)	1.01 (0.31, 3.28)	0.99
HTI (%)	8 (2.2)	11 (3.1)	0.74 (0.29, 1.91)	0.53
ECB (%)	2 (0.6)	3 (0.8)	0.62 (0.10, 3.86)	0.61
MEH (%)	1 (0.3)	1 (0.3)	1.55 (0.08, 31.60)	0.78
**Death**				
By day 7 (%)	14 (3.9)	13 (3.7)	1.21 (0.52, 2.80)	0.66
By day 90 (%)	57 (16.0)	57 (15.9)	1.03 (0.64, 1.64)	0.91
**Randomized** ** <6 h**				
*N* (%)	206/357 (57.7)	183/358 (51.1)		
**Primary outcome**				
mRS at day 90 [/6]–adjusted	3 [1,5]	3 [2,5]	0.81 (0.56, 1.18)	0.27
Unadjusted			0.84 (0.59, 1.19)	0.33
**Primary safety outcome**				
Intracranial bleeding (%)	9 (4.4)	14 (7.7)	0.60 (0.24, 1.48)	0.27
**Secondary safety outcomes**				
sICH (%)	3 (1.5)	4 (2.2)	0.74 (0.16, 3.52)	0.70
HTI (%)	5 (2.4)	10 (5.5)	0.46 (0.15, 1.41)	0.17
ECB (%)	1 (0.5)	2 (1.1)	0.35 (0.02, 5.79)	0.47
MEH (%)	0	0	–	–
**Death**				
By day 7 (%)	4 (2.0)	3 (1.7)	1.05 (0.21, 5.26)	0.95
By day 90 (%)	32 (15.5)	32 (17.5)	0.86 (0.44, 1.69)	0.67

*Data are number (%), odds ratio (OR), hazard ratio (HR) with 95% confidence intervals (CI) using ordinal or binary logistic regression*.

*CI, confidence interval; ECB, extracranial bleeding; GTN, glyceryl trinitrate; HR, Hazard ratio; HTI, haemorrhagic transformation of infarction; mRS, modified Rankin Scale; OR, odds ratio; sICH, symptomatic intracranial hemorrhage*.

*Adjusted for age, sex, baseline mRS, baseline NIHSS, baseline systolic BP, time to randomization, and trial*.

**Figure 2:**
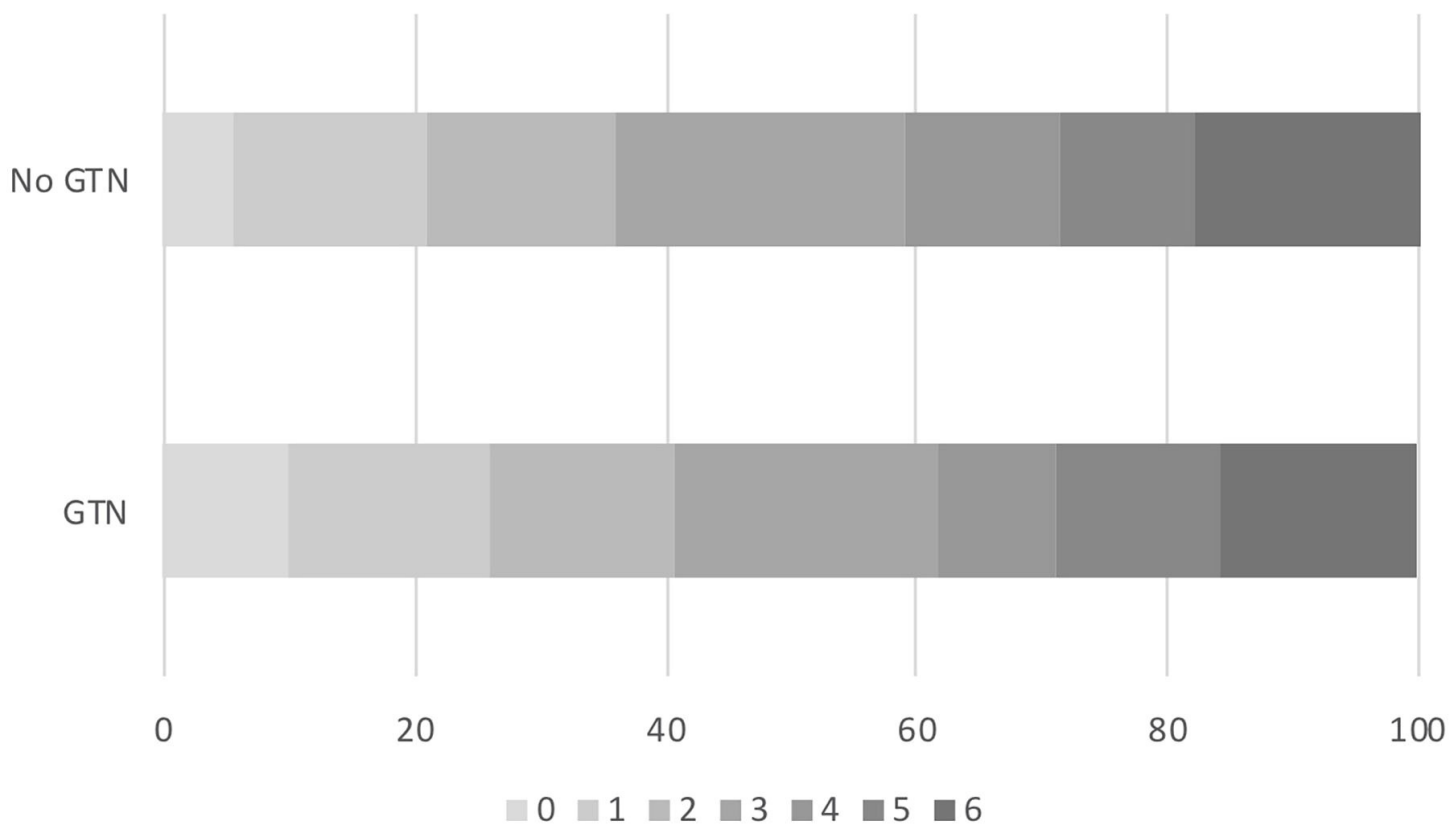
Acute ischaemic stroke patients who underwent reperfusion strategies and randomized to GTN vs. no GTN within 6 h of onset. Distribution of mRS at day 90 by GTN vs. no GTN: adjusted OR 0.81, 95% CI 0.56–1.18, *p* = 0.27. CI, confidence interval; GTN, glyceryl trinitrate; mRS, modified Rankin Scale; OR, odds ratio.

In those randomized to GTN or no GTN within 6 h of onset, there was a non-significant tendency toward improved 90 day functional outcome in both unadjusted and adjusted analyses: OR 0.81, 95% CI 0.56 to 1.18, *p* = 0.27 (Table 2, Figure 2). Similarly, there were numerically fewer intracranial bleeds, sICH, and HTI in those randomized to GTN than no GTN, but none of these analyses reached statistical significance. Rates of ECB and MEH, and death at day 7 and 90 did not differ between treatment groups (Table 2).

### Subgroup Analyses

**Figure 3:**
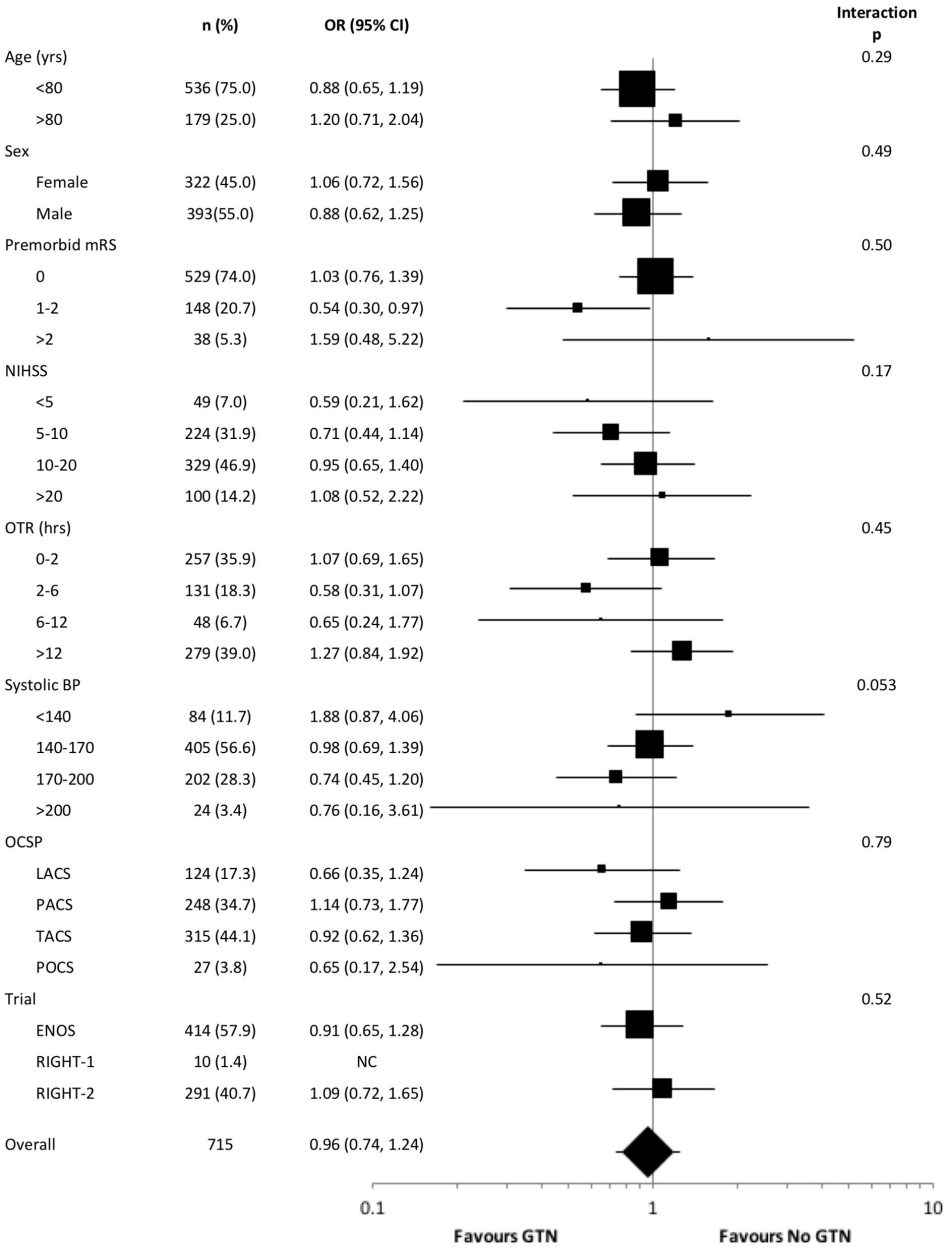
Forest plot of effect on mRS at day 90 in pre-defined subgroups for acute ischaemic stroke patients who underwent reperfusion strategies by GTN vs. no GTN. Unadjusted ordinal logistic regression for mRS at day 90 with interaction between subgroup and GTN vs. no GTN. BP, blood pressure; CI, confidence interval; ENOS, Efficacy of nitric oxide in stroke trial; GTN, glyceryl trinitrate; LACS, lacunar syndrome; mRS, modified Rankin scale; NIHSS, National Institutes for Health Stroke Scale; OCSP, Oxfordshire community stroke project; OTR, onset to randomization; PACS, partial anterior circulation syndrome; POCS, posterior circulation syndrome; RIGHT, Rapid intervention with glyceryl trinitrate in hypertensive stroke trial; TACS, total anterior circulation syndrome.

In an unadjusted ordinal logistic regression model there were no significant interactions noted between subgroups and randomization to GTN vs. no GTN (Figure 3). The baseline systolic BP subgroup interaction narrowly missed statistical significance (*p* = 0.053), with a suggestion that treatment with GTN in participants with increasing systolic BP may be associated with tendencies toward improved functional outcome at 90 days. Further, treatment with GTN in those with baseline systolic BP <140 mmHg may be associated with worse functional outcome (Figure 3).

## Discussion

In this individual patient data meta-analysis of trials assessing transdermal GTN vs. no GTN in acute ischaemic stroke patients who underwent reperfusion therapies (>99% thrombolysis), treatment with GTN was safe but did not influence functional outcome at 90 days. Bleeding complications after reperfusion therapies were infrequent and not influenced by GTN treatment overall. However, in those randomized to GTN vs. no GTN within 6 h of onset there was a tendency toward improved functional outcome at 90 days and reduced rates of intracranial haemorrhagic complications following reperfusion therapies, but these findings did not reach statistical significance. Randomization to GTN in those with higher systolic BP may be associated with a tendency toward improved functional outcome.

BP lowering in patients with acute ischaemic stroke is primarily reserved for patients with BP >185/110 mmHg who are otherwise eligible for thrombolysis (12). There is some evidence to suggest that in the context of large vessel occlusion, intensive lowering of elevated BP may be harmful (13). However, a *U*-shaped association with outcome was noted in the MR CLEAN registry with both high and low levels of baseline systolic BP being associated with worse clinical outcomes, whilst increasing systolic BP was associated with increased rates of sICH (14). Although there were too few thrombectomy patients in the current study to add to this evidence base, transdermal GTN was safe and did not worsen clinical outcomes nor affect the occurrence of haemorrhagic complications in a population predominantly treated with thrombolysis. This is in keeping with the overall results of prior GTN in acute stroke trials to date; GTN lowers BP, is safe but does not influence functional outcome at 90 days (7, 15). NO has significant antiplatelet properties, but NO donors may differ in their clinical antiplatelet effects: for example, GTN, an organic NO donor, may have antiplatelet properties *in vitro* (16) but did not *in vivo* in stroke patients (17); in contrast, sodium nitroprusside, an inorganic NO donor, has both *in vitro* and *in vivo* antiplatelet effects (18). Further, GTN may interrupt the early vasoconstrictory phase in hemorrhage (19), thus leading to more severe bleeding. In a subgroup analysis involving the intracerebral hemorrhage patients recruited in the RIGHT-2 trial, there was a tendency toward worse clinical outcomes in those randomized to GTN vs. no GTN (20). However, this was a small subgroup of the RIGHT-2 trial and the findings may represent undetected baseline imbalances. Reassuringly, in the present meta-analysis there were few bleeding events and we did not see any signal of increased haemorrhagic complications. Instead, there was a suggestion that GTN administered within 6 h of symptom onset in those undergoing reperfusion treatments may be associated with fewer bleeding complications, but this did not reach statistical significance.

The interaction between baseline systolic BP and treatment with GTN vs. no GTN narrowly missed statistical significance (*p* = 0.053), with increasing baseline BP associated with a tendency toward improved functional outcome in the presence of GTN. This may suggest that modest lowering of elevated BP in the context of thrombolysis may be beneficial, perhaps priming patients for earlier thrombolysis without the need for intravenous BP lowering, whilst also reducing the risk of potential haemorrhagic complications. This is line with the ENCHANTED-BP trial demonstrating less intracranial bleeding in those treated with intensive BP lowering vs. standard BP lowering, although there was no effect on functional outcome at 90 days (5). The present study also demonstrated that treatment with GTN in those with a baseline systolic BP <140 mmHg was associated with a tendency toward worse functional outcome at 90 days. BP lowering in this group may have compromised cerebral blood flow, extending the ischaemic core resulting in worse clinical outcomes. Therefore, this approach should be avoided in those with systolic BP <140 mmHg in the context of reperfusion therapies.

The strengths of this analysis include the ability to assess treatment effects at an individual patient level across the included trials and a high proportion of available outcome data. However, there are limitations. First, the timing of thrombolysis and thrombectomy was not recorded in the trials. In RIGHT and RIGHT-2, randomization to GTN vs. no GTN occurred in the ambulance prior to hospital and therefore reperfusion therapies. In ENOS, the relationship between time of randomized treatment administration and thrombolysis is less clear. We attempted to address this by assessing those participants randomized within 6 h of symptom onset separately, as in this group the randomized treatment is likely to have been received in close proximity to thrombolysis. Second, we do not have angiographic data on the proportion of patients with a confirmed large vessel occlusion at baseline, nor the recanalisation rate on any subsequent imaging. We were therefore unable to ascertain whether GTN was safe and efficacious in this population, or whether GTN had an effect based upon recanalisation status. Third, data regarding other BP lowering medications administered in relation to reperfusion therapies were not available. Such additional medication may have attenuated any treatment effect of GTN. Last, all included trials were performed by the same group and unfortunately included very few participants treated with thrombectomy. The ongoing MR ASAP trial in the Netherlands is assessing the use of a single GTN patch in the ambulance with a particular focus on patients with large vessel occlusions, adding vital data on this population (ISCRTN:99503308) (21).

In summary, this individual patient data meta-analysis has demonstrated that transdermal GTN is safe in patients with acute ischaemic stroke undergoing reperfusion therapies (>99% thrombolysis), but does not influence functional outcome at 90 days. GTN within 6 h of stroke onset in this setting was associated with tendencies toward improved functional outcome and fewer haemorrhagic complications. The timing of BP lowering in the context of reperfusion therapies warrants further investigation, particularly in regard to thrombectomy and recanalisation status, in ongoing and future studies.

## Data Availability Statement

The raw data supporting the conclusions of this article will be made available by the authors upon reasonable request.

## Author Contributions

JA performed the literature search, pooled analyses, statistical analysis and interpretation, manuscript drafting, and editing. LW performed pooling of databases, statistical interpretation, reviewed, and edited the manuscript. NS performed statistical interpretation, reviewed, and edited the manuscript. JW set up and co-ordinated all scan reading, including scan rating, training and data cleaning, reviewed and edited the manuscript. PB conceptualized the study, statistical interpretation, reviewed, and edited the manuscript, is corresponding author and has responsibility for submission. All authors contributed to the article and approved the submitted version.

## Conflict of Interest

PB is Stroke Association Professor of Stroke Medicine, is a National Institute of Health Research Emeritus Senior Investigator and was chief investigator for the ENOS, RIGHT, and RIGHT-2 trials. The remaining authors declare that the research was conducted in the absence of any commercial or financial relationships that could be construed as a potential conflict of interest.

## Abbreviations

ECB: Extracranial bleeding
ENOS: Efficacy of nitric oxide in stroke
HTI: transformation of infarction
MEH: Major extracranial hemorrhage
mRS: modified Rankin Scale
RIGHT: Rapid intervention with glyceryl trinitrate in hypertensive stroke trial
RIGHT-2: Rapid intervention with glyceryl trinitrate in hypertensive stroke trial-2
sICH: symptomatic intracranial hemorrhage.
