# Corrigendum: Intracranial Bleeding After Reperfusion Therapy in Acute Ischaemic Stroke Patients Randomized to Glyceryl Trinitrate vs. Control: An Individual Patient Data Meta-Analysis

**DOI:** 10.3389/fneur.2020.625572

**Published:** 2020-11-26

**Authors:** Jason P. Appleton, Lisa J. Woodhouse, Nikola Sprigg, Joanna M. Wardlaw, Philip M. Bath

**Affiliations:** ^1^Stroke Trials Unit, Division of Clinical Neuroscience, University of Nottingham, Nottingham, United Kingdom; ^2^Stroke, University Hospitals Birmingham National Health Service Foundation Trust, Birmingham, United Kingdom; ^3^Stroke, Nottingham University Hospitals National Health Service Trust, Nottingham, United Kingdom; ^4^Centre for Clinical Brain Sciences, University of Edinburgh, Edinburgh, United Kingdom

**Keywords:** bleeding, glyceryl trinitrate, ischaemic stroke, reperfusion, thrombolysis, thrombectomy, meta-analysis

Joanna M. Wardlaw was not included as an author in the published article. The corrected Author Contributions and Funding statements appear below.

## Author Contributions

JA performed the literature search, pooled analyses, statistical analysis and interpretation, manuscript drafting, and editing. LW performed pooling of databases, statistical interpretation, reviewed, and edited the manuscript. NS performed statistical interpretation, reviewed, and edited the manuscript. JW set up and co-ordinated all scan reading, including scan rating, training and data cleaning, reviewed and edited the manuscript. PB conceptualized the study, statistical interpretation, reviewed, and edited the manuscript, is corresponding author and has responsibility for submission. All authors contributed to the article and approved the submitted version.

## Funding

There was no specific funding for the present study. ENOS was funded by the UK Medical Research Council (G0501797) and BUPA foundation. RIGHT was funded by Nottingham University Hospitals NHS Trust (R&D Pump Priming Competition) and by the Division of Stroke, University of Nottingham. RIGHT-2 was funded by the British Heart Foundation (CS/14/4/30972). JW declares funding from the Scottish Imaging Network, A Platform for Scientific Excellence (SINAPSE) and the UK Dementia Research Institute which receives funding from the UK Medical Research Council, Alzheimer's Society and Alzheimer's Research UK. The funders were not involved in the study design, collection, analysis, interpretation of data, the writing of this article or the decision to submit it for publication.

The authors apologize for this error and state that this does not change the scientific conclusions of the article in any way. The original article has been updated.

