# Supplementary material for: Intracranial Bleeding After Reperfusion Therapy in Acute Ischaemic Stroke Patients Randomized to Glyceryl Trinitrate vs. Control: An Individual Patient Data Meta-Analysis
Source: Front Neurol. 2020 Oct 20;11:584038. doi: 10.3389/fneur.2020.584038 (PMC7606455; doi:10.3389/fneur.2020.584038)
Supplement: Supplementary file 1 [file Data_Sheet_1.PDF]

Supplementary Material for: Intracranial bleeding after reperfusion therapy in acute ischaemic stroke patients randomised to glyceryl trinitrate vs. control: an individual patient data meta-analysis

Supplementary Tables **2**

Supplementary Figures **6**

Supplementary Table 1: PRISMA checklist

Section/topic	#	Checklist item	Reported on page #
**TITLE**			
Title	1	Identify the report as a systematic review, meta-analysis, or both.	6
**ABSTRACT**			
Structured summary	2	Provide a structured summary including, as applicable: background; objectives; data sources; study eligibility criteria, participants, and interventions; study appraisal and synthesis methods; results; limitations; conclusions and implications of key findings; systematic review registration number.	2
**INTRODUCTION**			
Rationale	3	Describe the rationale for the review in the context of what is already known.	5, 6
Objectives	4	Provide an explicit statement of questions being addressed with reference to participants, interventions, comparisons, outcomes, and study design (PICOS).	6
**METHODS**			
Protocol and registration	5	Indicate if a review protocol exists, if and where it can be accessed (e.g., Web address), and, if available, provide registration information including registration number.	6
Eligibility criteria	6	Specify study characteristics (e.g., PICOS, length of follow-up) and report characteristics (e.g., years considered, language, publication status) used as criteria for eligibility, giving rationale.	6
Information sources	7	Describe all information sources (e.g., databases with dates of coverage, contact with study authors to identify additional studies) in the search and date last searched.	6
Search	8	Present full electronic search strategy for at least one database, including any limits used, such that it could be repeated.	6
Study selection	9	State the process for selecting studies (i.e., screening, eligibility, included in systematic review, and, if applicable, included in the meta-analysis).	6
Data collection process	10	Describe method of data extraction from reports (e.g., piloted forms, independently, in duplicate) and any processes for obtaining and confirming data from investigators.	6
Data items	11	List and define all variables for which data were sought (e.g., PICOS, funding sources) and any assumptions and simplifications made.	6, 7
Risk of bias in individual studies	12	Describe methods used for assessing risk of bias of individual studies (including specification of whether this was done at the study or outcome level), and how this information is to be used in any data synthesis.	6
Summary measures	13	State the principal summary measures (e.g., risk ratio, difference in means).	7
Synthesis of results	14	Describe the methods of handling data and combining results of studies, if done, including measures of consistency (e.g., I^2) for each meta-analysis.	7

Page 1 of 2

Section/topic	#	Checklist item	Reported on page #
Risk of bias across studies	15	Specify any assessment of risk of bias that may affect the cumulative evidence (e.g., publication bias, selective reporting within studies).	8
Additional analyses	16	Describe methods of additional analyses (e.g., sensitivity or subgroup analyses, meta-regression), if done, indicating which were pre-specified.	7
**RESULTS**			
Study selection	17	Give numbers of studies screened, assessed for eligibility, and included in the review, with reasons for exclusions at each stage, ideally with a flow diagram.	8
Study characteristics	18	For each study, present characteristics for which data were extracted (e.g., study size, PICOS, follow-up period) and provide the citations.	8
Risk of bias within studies	19	Present data on risk of bias of each study and, if available, any outcome level assessment (see item 12).	8
Results of individual studies	20	For all outcomes considered (benefits or harms), present, for each study: (a) simple summary data for each intervention group (b) effect estimates and confidence intervals, ideally with a forest plot.	8
Synthesis of results	21	Present results of each meta-analysis done, including confidence intervals and measures of consistency.	9
Risk of bias across studies	22	Present results of any assessment of risk of bias across studies (see Item 15).	8
Additional analysis	23	Give results of additional analyses, if done (e.g., sensitivity or subgroup analyses, meta-regression [see Item 16]).	9
**DISCUSSION**			
Summary of evidence	24	Summarize the main findings including the strength of evidence for each main outcome; consider their relevance to key groups (e.g., healthcare providers, users, and policy makers).	10
Limitations	25	Discuss limitations at study and outcome level (e.g., risk of bias), and at review-level (e.g., incomplete retrieval of identified research, reporting bias).	11
Conclusions	26	Provide a general interpretation of the results in the context of other evidence, and implications for future research.	11, 12
**FUNDING**			
Funding	27	Describe sources of funding for the systematic review and other support (e.g., supply of data); role of funders for the systematic review.	12

From: Moher D, Liberati A, Tetzlaff J, Altman DG, The PRISMA Group (2009). Preferred Reporting Items for Systematic Reviews and Meta-Analyses: The PRISMA Statement. PLoS Med 6(7): e1000097. doi:10.1371/journal.pmed1000097

For more information, visit: www.prisma-statement.org.

Supplementary Table 2: Risk of bias of included studies

Bias	Authors' Judgement	Comment
**ENOS**		
Random sequence generation (selection bias)	Low risk	Randomisation with minimisation using a centralised computer
Allocation concealment (selection bias)	Low risk	Online, secure website
Blinding (performance bias and detection bias)	Low risk	Treatment was single-blinded (participant)
Blinding of participants and personnel (performance bias)	Unclear risk	Participants blinded but not administering clinician as no placebo patches available
Blinding of outcomes assessment (detection bias)	Low risk	Central outcome assessments by assessors blinded to treatment group
Incomplete outcome data (attrition bias)	Low risk	No difference between treatment groups
Selective reporting (reporting bias)	Low risk	All pre-specified outcomes reported
Other bias	Low risk	None
**RIGHT**		
Random sequence generation (selection bias)	Low risk	Simple randomisation.
Allocation concealment (selection bias)	Low risk	After consent obtained, an opaque envelope with gauze dressing +/- GTN patch was opened
Blinding (performance bias and detection bias)	Low risk	Treatment was single-blinded (participant)
Blinding of participants and personnel (performance bias)	Unclear risk	Participants blinded but not administering clinician as no placebo patches available
Blinding of outcomes assessment (detection bias)	Low risk	Central outcome assessments by assessors blinded to treatment group
Incomplete outcome data (attrition bias)	Low risk	No difference between treatment groups
Selective reporting (reporting bias)	Low risk	All pre-specified outcomes reported

Other bias	Low risk	None
**RIGHT-2**		
Random sequence generation (selection bias)	Low risk	Computer-based randomisation with minimisation by ambulance station
Allocation concealment (selection bias)	Low risk	Sealed envelope containing GTN patches or Duoderm dressings within a sealed box also containing gauze to cover the patch
Blinding (performance bias and detection bias)	Low risk	Treatment was single-blinded (participant)
Blinding of participants and personnel (performance bias)	Unclear risk	Participants blinded but not administering clinician as no placebo patches available
Blinding of outcomes assessment (detection bias)	Low risk	Central outcome assessments by assessors blinded to treatment group
Incomplete outcome data (attrition bias)	Low risk	No difference between treatment groups
Selective reporting (reporting bias)	Low risk	All pre-specified outcomes reported
Other bias	Low risk	None

Supplementary Figure 1: PRISMA Flow Diagram

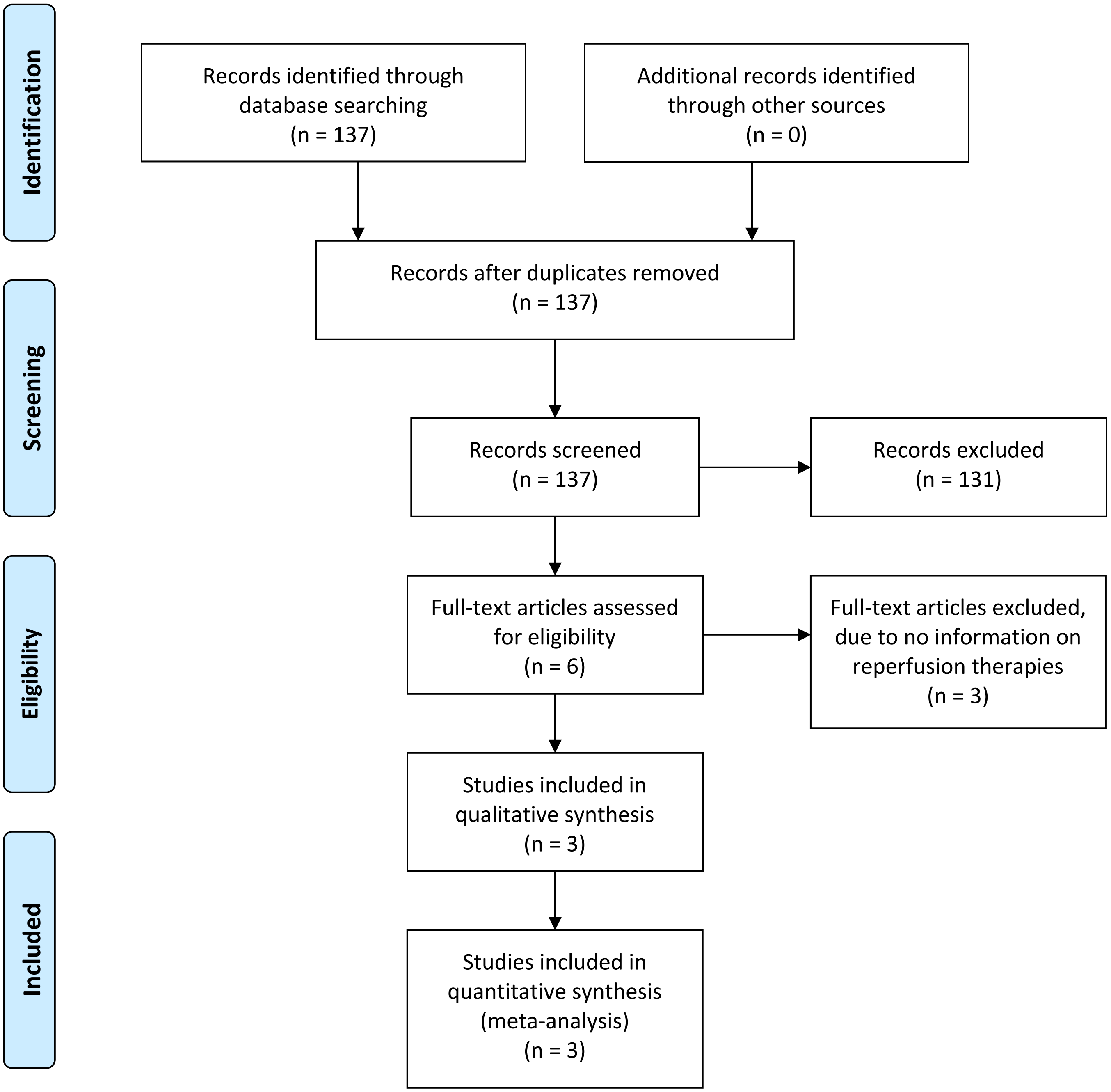

Supplementary Figure 2: Risk of bias graph. Presented as percentages across all included studies

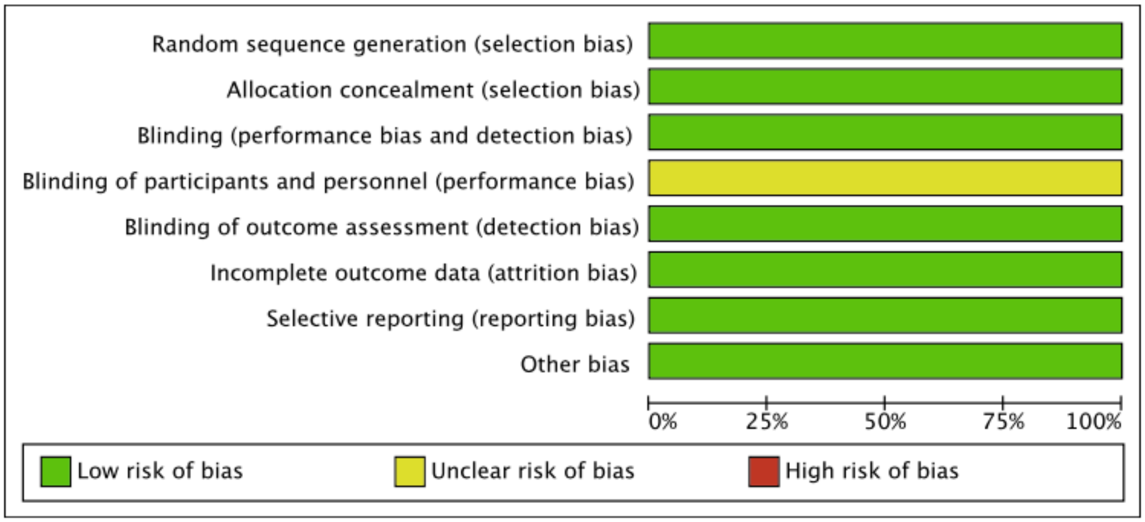

Supplementary Figure 3: Risk of bias summary. Risk of bias item for each included study.

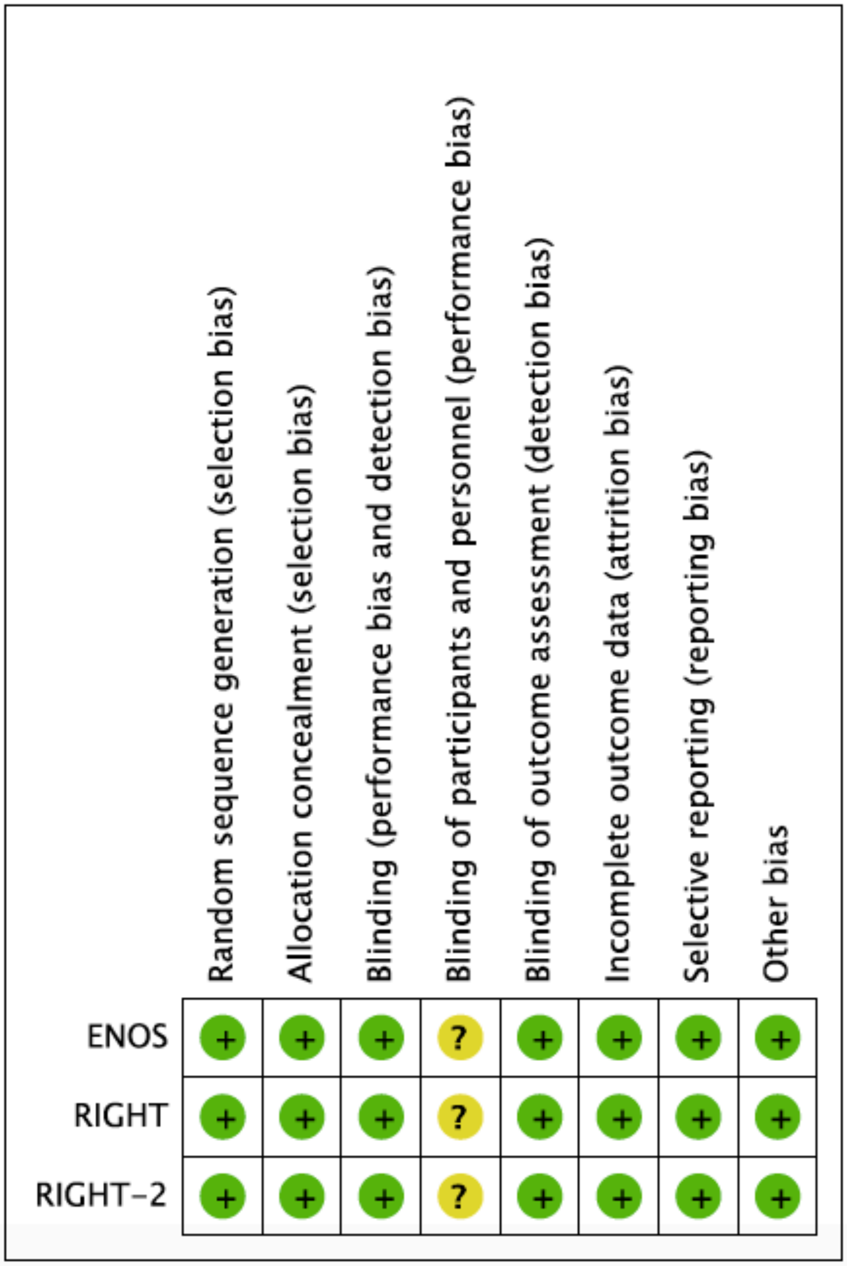

Green = low risk of bias. Yellow = unclear risk of bias. Red = high risk of bias.
